# ADCC-Inducing Antibody Trastuzumab and Selection of KIR-HLA Ligand Mismatched Donors Enhance the NK Cell Anti-Breast Cancer Response

**DOI:** 10.3390/cancers13133232

**Published:** 2021-06-28

**Authors:** Femke A. I. Ehlers, Nicky A. Beelen, Michel van Gelder, Tom M. J. Evers, Marjolein L. Smidt, Loes F. S. Kooreman, Gerard M. J. Bos, Lotte Wieten

**Affiliations:** 1 Department of Transplantation Immunology, Tissue Typing Laboratory, Maastricht University Medical Center+, 6229 HX Maastricht, The Netherlands; f.ehlers@maastrichtuniversity.nl (F.A.I.E.); nicky.beelen@maastrichtuniversity.nl (N.A.B.); t.m.j.evers@lacdr.leidenuniv.nl (T.M.J.E.); 2 Department of Internal Medicine, Division of Hematology, Maastricht University Medical Center+, 6229 HX Maastricht, The Netherlands; m.van.gelder@mumc.nl (M.v.G.); gerard.bos@mumc.nl (G.M.J.B.); 3 GROW—School for Oncology and Developmental Biology, Maastricht University, 6229 GT Maastricht, The Netherlands; m.smidt@mumc.nl (M.L.S.); loes.kooreman@mumc.nl (L.F.S.K.); 4 Medical Systems Biophysics and Bioengineering, Leiden Academic Centre for Drug Research, Faculty of Science, Leiden University, 2333 CC Leiden, The Netherlands; 5 Department of Surgery, Maastricht University Medical Center+, 6229 HX Maastricht, The Netherlands; 6 Department of Pathology, Maastricht University Medical Center+, 6229 HX Maastricht, The Netherlands

**Keywords:** alloreactive donor NK cells, HLA class I, antibody-dependent cellular cytotoxicity, breast cancer, tumor microenvironment

## Abstract

**Simple Summary:**

Natural killer (NK) cells are potent killers of tumor cells. Many tumors, including breast cancers, develop mechanisms to suppress anti-tumor immune responses, requiring the development of strategies to overcome suppression. Here, we tested a combination therapy that aims to (1) enhance NK cell activation and (2) reduce NK cell inhibition mediated by suppressive factors in tumors or in the tumor microenvironment. We cultured cell lines under hypoxia to mimic the tumor microenvironment or used patient-derived breast cancer cells that were primed by the patient’s tumor environment. Our results demonstrated that cytokine-activated NK cells remained active under hypoxia and that tumor-targeting antibodies enhanced the NK cell anti-breast cancer response. Moreover, we observed that NK cell suppression by inhibitory ligands on the tumor cells can be reduced by the selection of NK cell donors with NK receptors that are incompatible with these ligands. Collectively, we present two powerful strategies to enhance the NK cell responses against breast cancer.

**Abstract:**

Natural killer (NK)-cell-based immunotherapies are an attractive treatment option for cancer. We previously showed that alloreactive mouse NK cells cured mice of 4T1 breast cancer. However, the tumor microenvironment can inhibit immune responses, and these suppressive factors must be overcome to unfold the NK cells’ full anti-tumor potential. Here, we investigated the combination of antibody-dependent cellular cytotoxicity (ADDC) and the selection of KIR-HLA-ligand mismatched NK cells to enhance NK cell anti-breast cancer responses in clinically relevant settings. Donor-derived and IL-2-activated NK cells were co-cultured with patient-derived breast cancer cells or cell lines MCF7 or SKBR3 together with the anti-HER2 antibody trastuzumab. NK cells mediated anti-breast cancer cytotoxicity under normoxic and hypoxic conditions. Under both conditions, trastuzumab vigorously enhanced NK cell degranulation (CD107a) against HER2-overexpressing SKBR3 cells, but we observed a discrepancy between highly degranulating NK cells and a rather modest increase in cytotoxicity of SKBR3. Against patient-derived breast cancer cells, the anti-tumor efficacy was rather limited, and HLA class I expression seemed to contribute to inhibited NK cell functionality. KIR-ligand-mismatched NK cells degranulated stronger compared to the matched NK cells, further highlighting the role of HLA. In summary, trastuzumab and KIR-ligand-mismatched NK cells could be two strategies to potently enhance NK cell responses to breast cancer.

## 1. Introduction

Breast cancer is the most common cancer in women worldwide and remains a leading cause of death [1]. While newer therapies have improved survival rates over the past years, they still fail to cure metastatic disease. The effectiveness of standard therapies such as surgery, systemic therapy, and radiotherapy can be limited because tumor cells frequently develop resistance to therapy and subsequently progress [2]. Over the last years, immunotherapies, including monoclonal antibodies and cellular therapies, have emerged as promising treatment options. Trastuzumab (Herceptin) is a clinically approved antibody that targets human epidermal growth factor receptor 2 (HER2), a receptor that is overexpressed in 15–20% of breast cancer patients [3]. Next to direct anti-tumor effects, the mechanisms of trastuzumab include antibody-dependent cellular cytotoxicity (ADCC), which is mediated by CD16-expressing immune cells such as natural killer (NK) cells [4].

As NK cells are a crucial part of the first line of defense against tumors, they have gained increasing interest for cell-based immunotherapies, and ex vivo modifications can help to increase their effector functions in vivo in suppressive tumor microenvironments (TME) [5]. NK cells selectively kill tumor cells and, unlike T cells, do not require prior sensitization. They get activated when an excess of activation signals, such as stress signals, is received over inhibitory signals. The major inhibitory signals are mediated through human leukocyte antigens (HLA), which are expressed on all nucleated cells and bind to inhibitory killer-immunoglobulin-like receptors (KIRs) and NKG2A on NK cells. HLA ligands can be downregulated on tumor cells to escape T cells, but thereby tumor cells could become more susceptible to NK cells (missing-self hypothesis) [6]. HLA class I molecules are also critical for NK cell education, a process that is also known as NK cell licensing, and that requires the interaction between HLA ligands and the corresponding KIR or NKG2A receptors on the NK cell [7]. The more inhibitory receptors that find their ligand are expressed by NK cells, the higher the NK cell responsiveness against target cells, indicating that these receptors play a dual role in regulating NK cell effector functions by licensing NK cells on the one hand and inhibiting effector functions of previously licensed NK cells on the other hand [7].

In solid tumors, tumor-infiltrating NK cells are generally sparse, and they have been described as less cytotoxic than in healthy individuals [8]. To increase effectiveness, adoptive transfer of NK cells is tested. For this purpose, NK cells are either derived from the patient (autologous setting) or from healthy donors (allogeneic setting). Our group is focusing on developing effective donor-derived NK cell therapy for cancer. We have previously shown that murine NK cells from an HLA-haploidentical, alloreactive donor can cure mice of 4T1 breast cancer, while NK cells from a syngeneic donor failed to do so [9]. The concept of alloreactive NK cell donors, leading to an improved outcome compared to non-alloreactive donors, has previously been demonstrated in patients with acute myeloid leukemia that received haploidentical stem cell transplants [10]. NK cell alloreactive donors expressed licensed KIRs for which the corresponding HLA-ligands were missing in the recipient, similar to endogenous NK cells encountering tumor cells that downregulated HLA; these NK cells are also termed KIR-HLA ligand mismatched NK cells [11]. Alloreactive NK cells do not attack the recipient tissues, as activating ligands are absent on healthy cells [11].

Despite much progress with improving their anti-tumor responses, adoptive NK cells are not always effective yet. Tumors themselves can escape immune responses and develop resistance to therapy. In addition, the TME plays a crucial role in suppressing anti-tumor responses because it is frequently an environment with immunosuppressive factors, such as hypoxia, that can mediate inhibitory effects on immune cells including NK cells [12,13,14]. Hypoxic areas with a pO_2_ of 2.5 mm Hg (0.3% O_2_) or lower were detected in solid malignancies including breast cancer [15]. In another study, hypoxia, identified by HIF1α expression, was measured in about 40% of breast cancers and associated with poor survival [16]. Suppressive TME factors such as hypoxia must be overcome to unleash the break in NK cells and unfold their full anti-tumor potential. Such strategies include both potent activation of NK cells and minimizing NK cell inhibition. In a previous study, we observed that hypoxia reduced cytotoxicity and degranulation of unactivated NK cells and demonstrated that the oxygen levels during the kill assay were the most critical influencers of the response [12]. Importantly, we demonstrated that activation of the NK cells with IL-2 could almost completely restore the NK cell responses, illustrating that IL-2 is a potent activator of NK cells and that NK cells can mediate anti-tumor responses in a hypoxic environment when sufficiently activated [12]. Another strategy to better activate NK cells is the use of monoclonal antibodies that trigger ADCC via the CD16 NK cell receptor that binds to Fc-fragments of IgG antibodies. We and others showed that NK cells can mediate ADCC in a hypoxic environment against hematological tumors [17,18]. Trastuzumab may be a clinically applicable manner to enhance donor NK cell responses against breast cancer. In addition, HLA-mediated NK cell inhibition can be reduced by blocking the interactions of inhibitory receptors and their corresponding HLA ligands through blocking antibodies (e.g., anti-NKG2A antibody Monalizumab or anti-KIR antibody Lirilumab) or by selecting genetically different NK cell donors with a KIR–HLA mismatch [11,19].

In this study, we investigated whether the combination of ADDC-triggering and the selection of KIR–ligand mismatched NK cells can enhance the NK cell anti-tumor response to human breast cancer in clinically relevant settings. To address our research question, we used the anti-HER2 antibody trastuzumab and determined the cytotoxic potential of IL-2-activated, donor-derived NK cells in breast cancer models in the presence of hypoxia, an immunosuppressive factor frequently present in solid tumors. In addition, we evaluated the degranulation potency of KIR–ligand mismatched NK cells in this setting.

## 2. Materials and Methods

### 2.1. Cell Culture and Animals

The breast cancer cell line MCF7, purchased from ATCC, was cultured in an EMEM medium (ATCC, Manassas, VA, USA) supplemented with 10 µg/mL insulin, 10% fetal calf serum (FCS), 100 U/mL penicillin, and 100 µg/mL streptomycin (1% Pen/Strep, Thermo Fisher Scientific, Waltham, MA, USA). The breast cancer cell line SKBR3, purchased from DSMZ, was cultured in McCoy’s 5A medium (Gibco), supplemented with 20% FCS and 1% Pen/Strep. The HLA class I-negative cell line K562, purchased from ATCC, was used as a control cell line and cultured in IMDM medium (Gibco), supplemented with 10% FCS and 1% Pen/Strep. The cells were cultured at 37 °C in an incubator containing 21% O_2_ and 5% CO_2_. For hypoxia exposure, the cells were cultured at 37 °C in a hypoxic chamber containing 0.2% O_2_ and 5% CO_2_ (InvivO_2_ 1000, Ruskinn Technology Ltd., Bridgend, UK). NOD SCID gamma (NSG) mice were injected with 1 × 10^6^ MCF7 cells subcutaneously into the flank. The local animal ethical committee had approved the experiments. Primary tumors were harvested and dissociated into single-cell suspension using the Tumor Dissociation Kit human (Miltenyi Biotec, Bergisch Gladbach, Germany) together with gentleMACS Dissociator (Miltenyi). Dissociated tumor cells were frozen until assays were performed.

### 2.2. NK Cell Culture

NK cells were isolated from healthy anonymous buffy coats (Sanquin blood bank, Maastricht, The Netherlands). The use of buffy coats does not need ethical approval in the Netherlands under the Dutch Code for Proper Secondary Use of Human Tissue. NK cell donors with an HLA C1^+^ C2^+^ Bw4^+^ genotype and expression of KIR2DL1, KIR2DL2/3, and KIR3DL1 receptors were used to obtain NK cells licensed for all three KIRs. Peripheral blood mononuclear cells (PBMCs) were isolated by density gradient centrifugation using Lymphoprep (Axis-Shield, Dundee, Scotland). From the PBMCs, NK cells were obtained by negative selection, utilizing an NK cell isolation kit and the MACS separation column system (Miltenyi Biotec) according to the manufacturer’s protocol. The NK cells were subsequently activated with 1000 U/mL IL-2 (Proleukin, Novartis, Basel, Switzerland) and cultured overnight in RPMI-1640 medium (Gibco), supplemented with 10% FCS, 100 U/mL penicillin, and 100 µg/mL streptomycin at 37 °C in an incubator containing 21% O_2_ and 5% CO_2_. For some of the primary breast cancer cells, expanded NK cells were used as effector cells. NK cells were expanded from CD3-depleted PBMCs in SGCM medium supplemented with 10% FCS, 1% Pen/Strep, and 1000 U/mL IL-2. After 17 days of expansion, NK cells were frozen and, prior to the experiment, thawed and recovered overnight in the presence of IL-2.

### 2.3. Cytotoxicity Assay

The cytotoxic potential of NK cells against breast cancer cell lines was determined in flow-cytometry based assays. The target cells SKBR3, MCF7, and K562 were either labeled with CellTracker^TM^ CM-DiI Dye or with CellTracker^TM^ Deep Red Dye (both Thermo Fisher Scientific) and were incubated at 37 °C either with 21% O_2_ or with 0.2% O_2_. After 16 h incubation, the target cells were harvested and counted and 2 × 10^4^ cells were plated per well in a 96-well plate. The target cells were pre-incubated with 1 µg/mL trastuzumab (Roche, Basel, Switzerland) or with a culture medium, as a control, for 30 min. The hypoxia-exposed cells were kept at 0.2% O_2_ for all steps. IL-2-activated NK cells were harvested and washed before they were co-cultured with the target cells in a 1:1 or a 5:1 Effector:Target (E:T) ratio at 37 °C either with 21% O_2_ or with 0.2% O_2_. After 4 h of co-culture, plates were put on ice to stop the reaction. The cells were washed with PBS (Sigma-Aldrich, Munich, Germany) and stained for dead cells with Live/Dead^®^ Fixable Aqua Dead Cell Stain Kit (Thermo Fisher Scientific) for 30 min on ice. The assay was analyzed by flow cytometry. Specific cytotoxicity was calculated as follows: (% dead tumor cells − % spontaneous tumor cell death)/(100% − % spontaneous tumor cell death) × 100. Spontaneous tumor cell death in the presence of trastuzumab was used to calculate specific cytotoxicity in the conditions with trastuzumab.

### 2.4. CD107a Degranulation Assay

The target cell lines SKBR3, MCF7, and K562 were incubated for 16 h at 37 °C either with 21% O_2_ or with 0.2% O_2_ and subsequently harvested for the CD107a degranulation assay. In each well, 10^5^ target cells were plated and pre-incubated with 1 µg/mL trastuzumab (Roche) or, as a control, with a culture medium for 30 min. IL-2-activated NK cells were harvested, washed, and subsequently co-cultured with the target cells in a 1:1 E:T ratio at 37 °C either with 21% O_2_ or with 0.2% O_2_. To each well, 5 µL of CD107a-Horizon V450 antibody (Miltenyi) was added. After 1 h of co-culture, Monensin (BD Biosciences, San Jose, CA, USA) was added to prevent reinternalization of CD107a, and after another 3 h of co-culture, the plates were put on ice to stop the reaction. The cells were washed with PBS and first stained with Live/Dead^®^ Fixable Aqua Dead Cell Stain Kit (Thermo Fisher Scientific) for 30 min on ice before surface staining with the following antibodies was performed for 30 min on ice: anti-CD3-APC-Vio770 (BW264/56), anti-CD56-PerCP-Vio700 (REA196), anti-KIR2DL1-APC (143211), anti-KIR2DL2/3-PE (DX27), anti-KIR3DL1-FITC (DX9), and anti-NKG2A-PE-Vio770 (REA110). The assay was analyzed by flow cytometry.

### 2.5. KIR-Ligand Mismatched and Matched NK Cells

The HLA class I genotype of SKBR3 and MCF7 was determined by Luminex-SSO. The genotype for SKBR3 cells was HLA C1^+^ C2^−^ Bw4^−^, and the genotype for MCF7 cells was HLA C1^−^ C2^+^ Bw4^+^. Flow cytometry was performed to determine the phenotypic expression of HLA-C and Bw4 as described below. The matched and mismatched NK cell populations were identified based on the genotypic expression of HLA C1, HLA C2, and HLA Bw4, as well as the phenotypic expression of Bw4. For SKBR3, KIR-ligand-matched NK cells were KIR2DL2/3+ NK cells, and KIR-ligand-mismatched NK cells were KIR2DL1+, KIR3DL1+, and KIR2DL1+ KIR3DL1+ double-positive NK cells. For MCF7, KIR-ligand-matched NK cells were KIR2DL1+ NK cells, and KIR-ligand-mismatched NK cells were KIR2DL2/3+, KIR3DL1+, as well as KIR2DL2/3+ KIR3DL1+ double-positive NK cells.

### 2.6. Generation of F(ab’)2 Fragment

The Pierce F(ab’)_2_ Preparation Kit (Thermo Fisher Scientific) was used according to the manufacturer’s protocol to generate an F(ab’)_2_ fragment of the trastuzumab antibody. The following secondary antibody was used to stain for the F(ab’)_2_ fragment or trastuzumab: Alexa Fluor 647 AffiniPure F(ab’)_2_ Fragment Goat Anti-Human IgG, F(ab’)_2_ fragment specific (Jackson ImmunoResearch, Cambridgeshire, UK).

### 2.7. Primary Human Breast Cancer Cells

Primary human breast cancer tissue was obtained from the Maastricht Pathology Tissue Collection. Collection, storage, and use of tissue and patient data were performed in agreement with the “Code for Proper Secondary Use of Human Tissue in the Netherlands” and have been approved by the local ethics committee. The tissue was immediately stored in MACS Tissue Storage Solution (Miltenyi) until processing. To dissociate single cells, the Tumor Dissociation Kit human (Miltenyi) was used together with a gentleMACS Dissociator (Miltenyi) according to the manufacturer’s instructions. Subsequently, the cell suspension was enriched for tumor cells by negative selection using the Tumor Cell Isolation Kit, human (Miltenyi). Tumor cells were identified by PanCytokeratin-AF488 (C11, ThermoFisher) and the purity of tumor cells was at least 70% PanCK^+^ cells, with one exception of 44% PanCK^+^ cells. For cytotoxicity and CD107a assays, either freshly isolated or IL-2-expanded NK cells were used, and co-cultures were performed for a duration of 16 h.

### 2.8. Flow Cytometry

To determine HER2 and HLA surface expression, SKBR3 and MCF7 cells were stained with Live/Dead^®^ Fixable Aqua Dead Cell Stain Kit (Thermo Fisher Scientific) for 30 min on ice, followed by staining with HER2-APC (Neu24.7, BD), HLA-C-PE (DT9, BD), HLA-Bw4-PEVio770 (REA274, Miltenyi), HLA-E-PE (3D12, Thermo Fisher Scientific), HLA-ABC-APC (G46-2.6, BD), or HLA-ABC-PE (REA230, Miltenyi), or matched isotype controls for 30 min on ice. All analysis by flow cytometry was performed with BD FACS Canto II. Data were analyzed with FlowJo v10.6.1 64-bit software, (TreeStar, Ashland, OR, USA).

### 2.9. Statistics

The statistical analysis was performed with GraphPad Prism 8.4.3 software (Graphpad Software, San Diego, CA, USA) using paired, non-parametric *t*-tests (Wilcoxon matched-pairs signed-rank test).

## 3. Results

### 3.1. IL-2 Activated NK Cells Mediate Anti-Breast Cancer Responses under Hypoxia and Maintain the Potential to Mediate Trastuzumab-Induced ADCC

**Figure 1:**
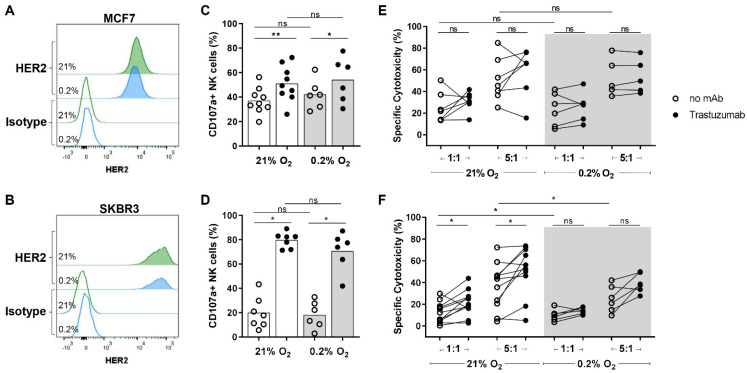
NK cell efficacy against non-amplified and HER2 amplified cell lines with or without trastuzumab under normoxia (21% O_2_) and hypoxia (0.2% O_2_). (**A**,**B**) HER2 surface expression levels were determined by flow cytometry on non-amplified MCF7 cells (**A**) and HER2-amplified SKBR3 cells (**B**). (**C**–**F**) For both degranulation (CD107a) and cytotoxicity assays, IL-2-activated NK cells were co-cultured with the target cells MCF7 or SKBR3 for 4 h either with 21% O_2_ or 0.2% O_2_ and analyzed by flow cytometry. CD107a assays were performed in 1:1 E:T ratios and % of CD107a^+^ NK cells are shown per donor against MCF7 (**C**) or SKBR3 (**D**) with the bar height indicating the mean. Cytotoxicity assays were done in 1:1 or 5:1 E:T ratios and dead target cells are shown as % of specific cytotoxicity for MCF7 (**E**) and SKBR3 (**F**). The donors from Figure 2 are included in the conditions with 21% O_2_ of (F). Each dot represents the average of duplicates from one NK cell donor. * *p* < 0.05, ** *p* < 0.01, ns = not significant.

Since hypoxia can have inhibitory effects on NK cells and is frequently observed in breast cancer [14,15], we investigated the influence of hypoxia on NK cell anti-tumor responses either in the presence or absence of trastuzumab, an ADCC triggering therapeutic antibody. We used the HER2-non-amplified cell line MCF7 and the HER2-amplified cell line SKBR3 in NK cell functional assays together with trastuzumab. First, the influence of hypoxia on HER2 surface expression was detected by flow cytometry. Both cell lines expressed HER2 but the expression level of MCF7 was around 8-fold lower than of SKBR3, confirming their gene amplification status and hypoxia (0.2% O_2_) did not influence HER2 expression of both cell lines (Figure 1A,B).

To determine the influence of hypoxia on NK cell degranulation, either or not in the presence of trastuzumab, CD107a assays were performed. The cell lines were cultured either under normoxia or hypoxia for 16 h before IL-2-activated NK cells were added for a 4 h co-culture period. To confirm that the NK cells from all donors were highly activated and functional, K562 cells were included as control target cells against which NK cells from all donors could strongly degranulate and mediate cytotoxicity (Figure S1). With MCF7 as target cells under normoxic conditions, the percentage of degranulating NK cells ranged from 20% to 56% without trastuzumab and from 26% to 72% with trastuzumab. The average degranulation increased from 37% without to 52% with trastuzumab (Figure 1C). Exposure of tumor cells to hypoxia did not reduce the degranulation of NK cells against MCF7 or the NK cell potentiating effect of trastuzumab (Figure 1C). Against the HER2-amplified SKBR3 cells, an average of 20% of NK cells degranulated under normoxia, and the addition of trastuzumab highly increased the percentage of degranulating NK cells to 80%, demonstrating vigorous degranulation in all donors (Figure 1D). Compared to normoxia, NK cell degranulation against SKBR3 was around 10% lower under hypoxic conditions, but this did not reach statistical significance, illustrating that degranulation levels of activated NK cells were not severely impaired by low oxygen (Figure 1D).

To assess the actual cytotoxic potential of NK cells in the presence of hypoxia and the ADCC-response mediated by trastuzumab, we performed 4 h cytotoxicity assays with IL-2-activated NK cells in different E:T ratios. Under normoxia, 25% of MCF7 cells were killed by NK cells in a 1:1 E:T ratio. As expected, the level of natural cytotoxicity was dependent on the E:T ratio and increased to an average of 52% in a 5:1 E:T ratio (Figure 1E). In line with NK cell degranulation, the cytotoxic capacity of MCF7 was not reduced by exposure to hypoxia (Figure 1E). With trastuzumab, cytotoxicity against the non-amplified MCF7 cells was increased in some donors, while it was decreased in other donors compared to natural cytotoxicity. Overall, trastuzumab did not enhance the average NK cell-mediated cytotoxicity against MCF7 under normoxic or hypoxic conditions (Figure 1E). The HER2-amplified SKBR3 cell line was more resistant to NK cell cytotoxicity. Under normoxic conditions, natural cytotoxicity ranged from 0–30% with an average of 11%, which was increased to 36% with a 5:1 E:T ratio (Figure 1F). The addition of trastuzumab increased the cytotoxicity in 9 out of 11 donors under normoxia and 5 out of 6 donors under hypoxia in both 1:1 and 5:1 E:T ratios. Although an ADCC effect by trastuzumab was observed, it was surprisingly small compared to the large increase in NK cell degranulation by trastuzumab. Increasing the concentration of trastuzumab up to 32 µg/mL did not further enhance the binding of the antibody to SKBR3 target cells (Figure S2A) or the ADCC response (Figure S2C). Compared to normoxia, NK-cell-mediated cytotoxicity was slightly reduced under hypoxic conditions, independent of trastuzumab and most evident in the 5:1 E:T ratio (36% under normoxia reduced to 25% under hypoxia) (Figure 1F).

**Figure 2:**
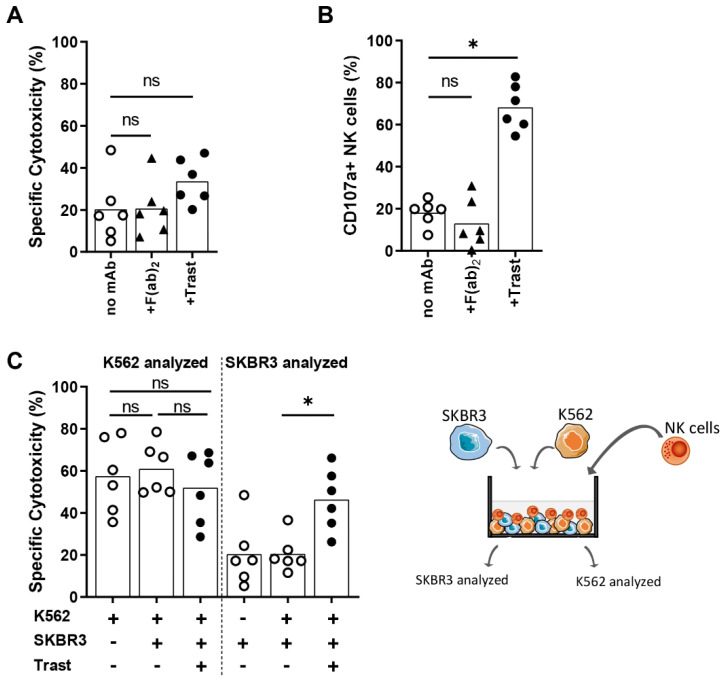
NK cell efficacy against the target cells SKBR3 in the presence of an F(ab)_2_ fragment of trastuzumab. SKBR3 and K562 target cells were labeled with different dyes and co-cultured with IL-2-activated NK cells in a 1:1 E:T ratio with trastuzumab or with a F(ab)_2_ fragment of trastuzumab for 4 h with 21% O_2_. Flow cytometry was used to analyze specific cytotoxicity of tumor cells (**A**,**C**) and, in separate assays, degranulation of NK cells (**B**). (**C**) Target cells K562 and SKBR3 were combined in one well with or without trastuzumab and analyzed for specific cytotoxicity of K562 and SKBR3. The schematic setup is depicted on the right, and the full gating strategy is shown in Figure S3. Each dot represents one NK cell donor, and the average of duplicates is shown per donor with the bars indicating the mean. Data from three donors of (**A**) are also used in Figure 1F. * *p* < 0.05, ns = not significant.

We observed a large discrepancy between the level of trastuzumab-induced NK cell degranulation and the level of trastuzumab-induced killing of HER2-positive target cells. Because of this observation and because tumors can develop resistance to the direct cytotoxic effects of trastuzumab [18], we investigated whether the binding of trastuzumab to SKBR3 had induced resistance of SKBR3 cells to the cytotoxic machinery of NK cells. To do this, the cytotoxicity assays were performed with an F(ab)_2_ fragment of trastuzumab, which can bind to HER2, but, due to the absence of an Fc-part, does not engage CD16 on the NK cell and therefore lacks the ADCC potentiating effects. We observed that binding of the F(ab)_2_ fragment to SKBR3 did not affect the killing of SKBR3 cells by NK cells (Figure 2A) or the level of NK cell degranulation (Figure 2B) as compared to the control condition without F(ab)_2_. This illustrates that the binding of trastuzumab to the HER2 antigen did not result in tumor cell resistance to NK cells. To test if the relatively low levels of ADCC were caused by trastuzumab-mediated inhibition of NK cell cytotoxicity, cytotoxicity assays were performed with HER2-overexpressing SKBR3 cells and HER2-negative K562 cells combined as target cells in one well (Figure 2C, gating strategy in Figure S3). This revealed that NK cells killed K562 equally well when K562 cells were cocultured with SKBR3 cells, either with trastuzumab or not (Figure 2C).

Taken together, these results imply that IL-2-activated NK cells remained cytotoxic against the tested breast cancer cell lines under hypoxia. Moreover, despite the relatively small increase in killing of target cells, trastuzumab could potentiate the NK cell anti-tumor response by strongly enhancing NK cell degranulation under normoxic and hypoxic conditions, especially against HER2 overexpressing target cells.

### 3.2. NK Cell Anti-Tumor Efficacy against Primary Breast Cancer Cells Is Tumor Dependent and May Be Related to Tumor Cell Expression of HLA Class I

**Figure 3:**
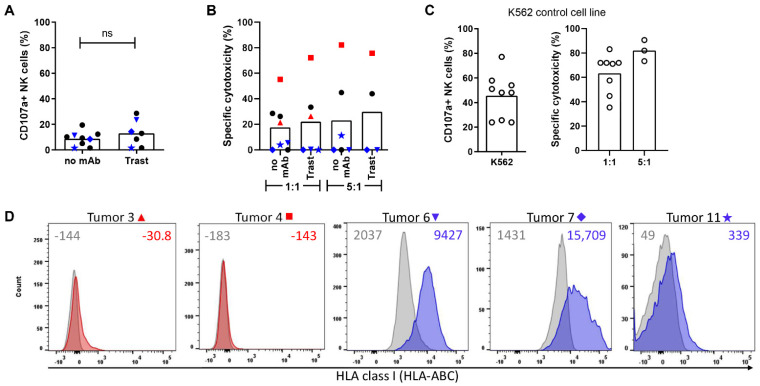
NK cell degranulation and cytotoxic potential against HER2 non-amplified primary breast cancer. (**A**,**B**) Patient-derived breast cancer cells were dissociated to single cells and incubated with or without trastuzumab for 30 min and subsequently co-cultured with IL-2-activated NK cells in 1:1 or 5:1 E:T ratios for 16 h at 21% O_2_ and analyzed by flow cytometry. (**A**) Degranulating NK cells are shown as percentage CD107a^+^ NK cells, and each symbol (black and blue) represents one tumor sample; the blue symbols correspond to the colors of the histograms in (**D**). (**B**) Dead tumor cells are shown as specific cytotoxicity. The bar graphs indicate the mean, while each dot represents an individual breast cancer specimen. The red and blue symbols correspond to the histograms in (**D**). (**C**) K562 cells were included as a control in CD107a and cytotoxicity assays to show that NK cells were potent killers. (**D**) Histograms show HLA class I staining of breast cancer cells (red for samples with kill, blue for samples with low kill), and grey histograms show isotype controls. Tumors 3 and 4 (red symbols) were not available for CD107a assays in A. Median fluorescent intensity is depicted for each staining. ns = not significant.

In addition to hypoxia, the TME consists of many other factors that can directly or indirectly suppress the NK cell anti-tumor response. To study NK cell efficacy against primary breast tumors derived from and potentially influenced by the clinically relevant TME in the patient, we set up a model utilizing human primary breast cancer cells that were isolated from patients. Leftover breast cancer material was included independently of the breast cancer subtype, but all samples were HER2-negative (non-amplified). Patients with HER2^+^ breast cancer are usually treated with neoadjuvant therapy with very good response, and therefore we could not obtain sufficient remaining material from HER2^+^ tumors to perform the assays. Where cell numbers allowed, trastuzumab was included to test if the level of HER2 expression on HER2 non-amplified breast cancer is sufficient to enhance NK cell activation. The patient-derived tumor tissues were dissociated into single-cell suspensions and subsequently enriched for tumor cells to specifically study the interaction of donor NK cells with tumor cells. Degranulation assays (CD107a) or cytotoxicity assays with the primary tumor cells as targets were performed by co-culturing these breast cancer cells with IL-2-activated NK cells as effector cells for 16 h. With an average of 10%, the overall NK cell degranulation against primary human breast cancer cells was rather low, and trastuzumab did not strongly increase NK cell degranulation against these HER2 non-amplified targets (Figure 3A). These data corresponded to our cell line data, indicating that HER2 expression as detected on non-amplified cells is not sufficient to trigger a strong increase in NK cell degranulation.

When analyzing the cytotoxic capacity of NK cells against the breast cancer cells, we observed a large variation between samples ranging from 0% to 60% specific cytotoxicity with 1:1 Effector:Target (E:T) ratios and from 0% to 80% in 5:1 E:T ratios (Figure 3B). In line with its effect on NK cell degranulation, trastuzumab did not mediate a clear ADCC effect (Figure 3B). K562 cells were included as a control to ensure that all NK cells were potent killers and, as expected, NK cells from all donors degranulated strongly and killed K562 cells (Figure 3C). Because HLA class I is an important inhibitory ligand for NK cells and its expression is frequently downregulated in breast cancer, either partially or completely [19], we stained for HLA class I expression on five tumors where we retrieved sufficient single cells to test the contribution of HLA class I to NK cell susceptibility. Two of those tumors were killed by NK cells (red histograms, corresponding to red symbols) and three were resistant to NK cell-mediated killing, resulting in low to no kill (blue histograms, corresponding to blue symbols) (Figure 3B). The tumors susceptible to NK cell-mediated killing (red symbols) expressed HLA class I at a very low level or not at all. Due to the low yield of tumor cells, we could not perform CD107a assays with the samples depicted in red symbols. The tumors resistant to NK cells (blue symbols) expressed HLA class I (Figure 3D). Our data illustrate that primary breast cancer cells can be relatively resistant to NK cells. Although the relative contribution of other factors, including both activating and inhibitory ligands, needs to be addressed in more detail, our data also suggest that HLA class I expression is associated with NK cell resistance.

### 3.3. KIR Ligand Mismatched NK Cells Degranulated More Vigorously Than Their HLA-Matched Counterparts, While Trastuzumab Activated All NK Cell Subsets

**Figure 4:**
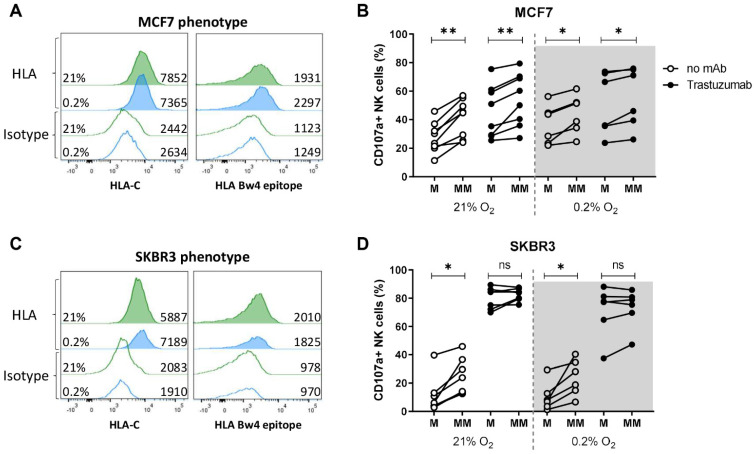
NK cell degranulation of KIR-ligand-matched and KIR-ligand-mismatched NK cells in response to breast cancer cells under normoxia or hypoxia with or without trastuzumab. (**A**,**B**) MCF7 and SKBR3 cells were exposed to 21% O_2_ or 0.2% O_2_ for 16 h and stained for HLA surface expression by flow cytometry. Histograms from normoxic conditions are indicated in green, with hypoxic conditions in blue. (**C**,**D**) Degranulation assays were performed by co-culturing NK wells either with MCF7 or SKBR3 target cells with or without trastuzumab for 4 h at 21% O_2_ or 0.2% O_2_. Based on the KIR expression of NK cells and HLA expression of target cells, KIR-ligand-matched (M) and -mismatched (MM) NK cell subsets were analyzed and the percentage of degranulating NK cells is shown per subset as % CD107a^+^ NK cells. Each dot represents the average of duplicates from one NK cell donor. * *p* < 0.05, ** *p* < 0.01, ns = not significant.

To further investigate the functional relevance of HLA class I as an inhibitor for NK cell anti-breast cancer responses under hypoxic conditions and in combination with trastuzumab, we investigated the degranulation level of NK cells that encounter their HLA class I ligand (KIR-ligand-matched) versus NK cells that do not encounter their ligand (KIR-ligand-mismatched). Such mismatch situations can occur either when endogenous NK cells encounter tumor cells that downregulated HLA or in the situation where patients receive donor NK cells that were selected based on the presence of a KIR-ligand mismatch to further potentiate NK cell anti-tumor responses. To enable analysis of the KIR-ligand-matching status, breast cancer cell lines were geno- and phenotyped for HLA class I. The genotype of MCF7 cells was determined as HLA C1^−^, C2^+^, and Bw4^+^ and the genotype of SKBR3 as HLA C1^+^, C2^−^, and Bw4^−^. HLA-C expression was confirmed by surface staining in both cell lines (Figure 4A,B). Despite the Bw4^+^ genotype on MCF7, the Bw4 molecule was not clearly expressed on the cell surface (Figure 4A). The phenotypic expression of HLA-C and Bw4 was not altered by exposure to hypoxia in either of the two cell lines (Figure 4A,B). To determine the KIR-ligand-matched and mismatched NK cell subsets, NKG2A^−^ NK cells that were single positive for either KIR2DL1, KIR2DL2/3, or KIR3DL1 were selected during analysis. Subsequently, the cell populations were grouped as the matched NK cell subset when the ligand was present on the target cells or as the mismatched NK cell subset when the corresponding HLA ligand was absent.

The KIR-ligand-matched NK cell subset for MCF7 was KIR2DL1^+^, while the KIR-ligand-mismatched NK cells for MCF7 were KIR2DL2/3^+^, KIR3DL1^+^, or KIR2DL2/3^+^ KIR3DL1^+^ double-positive cells. We included KIR3DL1 in the mismatched subset because of the very low Bw4 expression on MCF7. KIR-ligand-mismatched NK cells of all donors degranulated stronger against MCF7 cells than their KIR-ligand-matched counterparts (degranulation of seven out of eight donors under normoxia and five out of six donors under hypoxia increased at least 5%, Figure 4B). In the presence of trastuzumab, when total NK cell degranulation was slightly enhanced, the mismatched NK cell subset degranulated stronger than the matched NK cell subset in both normoxic and hypoxic conditions (Figure 4B, Figure S4A). With SKBR3 as target cells, the KIR-ligand-matched NK cell subset expressed KIR2DL2/3 and the KIR-ligand-mismatched subset expressed KIR2DL1, KIR3DL1, or a combination thereof. Compared to the matched population, NK cell degranulation of the KIR-ligand-mismatched population was enhanced more than 5% in seven out of seven donors under normoxia and five out of six donors under hypoxia (Figure 4D, Figure S4B). Trastuzumab by itself induced vigorous degranulation against SKBR3 in all NK cell subsets under normoxia and under hypoxia (Figure 4D). Due to the high degranulation, a potential additional effect of KIR-ligand mismatching with trastuzumab was not detected (Figure 4D). NK cell degranulation of each subset against the HLA-deficient line K562 was analyzed to control for intrinsic difference between the subsets. A minor increase in degranulation from matched to mismatched NK cell subsets was observed against K562 cells; however, the increase was less consistent and much lower than the increase observed with MCF7 and SKBR3 as target cells (Figure S5). Altogether, these results demonstrated that KIR-ligand-mismatched NK cells degranulated more potently than their matched counterparts, indicating that HLA class I is a relevant inhibitory factor in normoxic as well as in hypoxic conditions, while trastuzumab alone resulted in strong NK cell degranulation of all subsets against HER2-amplified targets.

**Figure 5:**
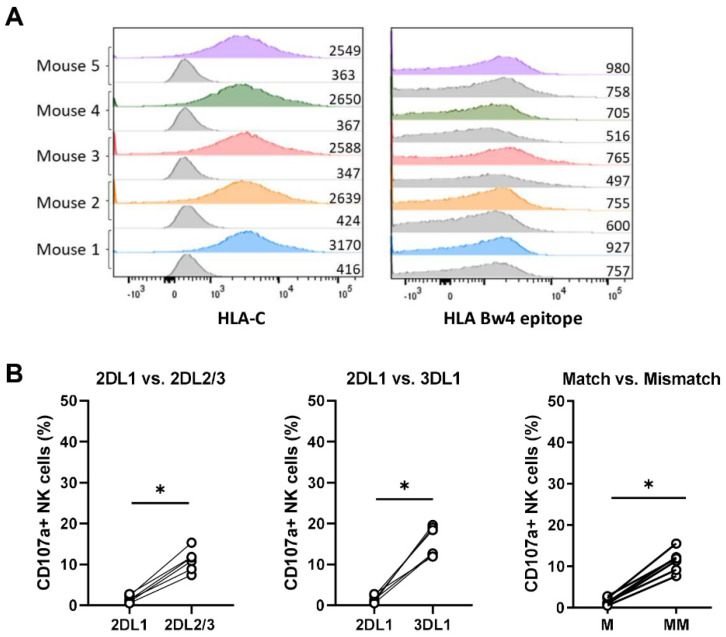
NK cell degranulation of KIR-ligand-matched and -mismatched NK cells in response to MCF7 grown in vivo. MCF7 tumors that were grown in mice were harvested and dissociated into single-cell suspension before performing HLA staining with *n* = 5 tumors (**A**) or 4 h CD107a assays with *n* = 6 tumors (**B**). Within the CD107a assay, degranulation is shown for each receptor (left and middle graph) and also as KIR-ligand-matched (M, 2DL1) vs. -mismatched (MM, 2DL2/3 and 3DL1) NK cell subsets as % CD107a^+^ NK cells. Each dot represents a tumor isolated from one mouse and the average of duplicates is depicted. * *p* < 0.05.

HLA expression levels are dynamically regulated by context-specific factors such as interferons [20] or 3D cell growth [21]. We therefore used a mouse model with MCF7 cells growing in immunodeficient mice to better mimic the three-dimensional growth of breast cancer in a TME. From these in vivo grown MCF7 tumors, single-cell suspensions were obtained and HLA class I expression levels and their impact on NK cell degranulation were evaluated in ex vivo CD107a assays. HLA-C expression levels on in vivo grown MCF7 cells were higher compared to MCF7 cultured in vitro (seven-fold expression vs. three-fold expression compared to isotype control in MCF7 ex vivo (Figure 5A) vs. in vitro (Figure 4A)), which underlines the importance of the environmental context. The KIR-HLA-ligand-matched KIR2DL1^+^ single-positive NK cell subset did not degranulate against those mouse-derived tumor cells, while the mismatched KIR2DL2/3^+^ and KIR3DL1^+^ single-positive populations degranulated (Figure 5B). Despite the Bw4-positive genotype, Bw4 expression remained negative on MCF7 ex vivo (Figure 5A). Consistent with this Bw4-negative phenotype, the corresponding KIR3DL1 receptor degranulated stronger than the matched KIR2DL1 against MCF7 ex vivo (Figure 5B). Overall, the ex vivo data confirm our in vitro results that stronger degranulation was observed in the KIR-ligand-mismatched NK cell subsets.

### 3.4. NK Cell Subsets Expressing NKG2A Degranulate More Potently against HLA-E Negative Tumors Than NK Cell Subsets without NKG2A

**Figure 6:**
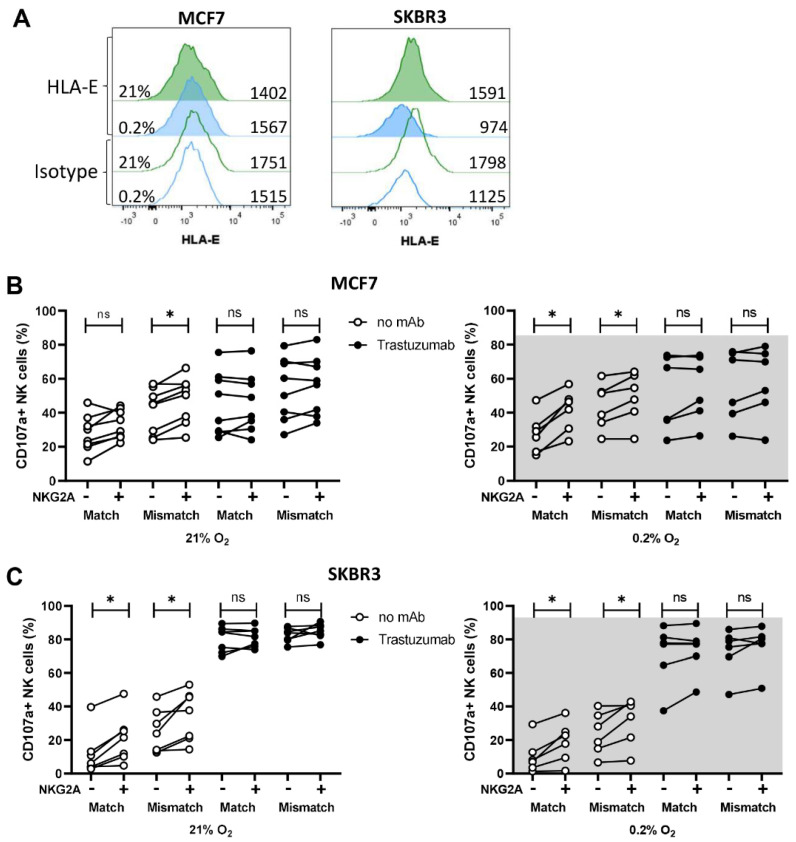
NK cell degranulation of the NKG2A^+^ subsets in comparison to the NKG2A^−^ subsets is slightly enhanced. (**A**) HLA-E expression of MCF7 and SKBR3 was determined by flow cytometry. (**B**,**C**) For degranulation assays, NK cells were co-cultured either with MCF7 or SKBR3 targets cells with or without trastuzumab for 4 h at 21%O_2_ or 0.2% O_2_. By flow cytometry analysis, NK cells were grouped in NKG2A^−^ and NKG2A^+^ subsets (indicated by − and + below graphs) and further divided into KIR-ligand-matched and -mismatched subsets based on their KIR expression. For each subset, NK cell degranulation (CD107a in %) is depicted in response to MCF7 (**B**) or SKBR3 (**C**). Each dot represents one NK cell donor and the average of duplicates. * *p* < 0.05, ns = not significant.

NKG2A has, similar to KIR, a dual role in regulating NK cell functions; on the one hand by licensing NK cells, whereby NK cells can become more effective, and on the other hand by mediating inhibitory signals upon binding its ligand HLA-E. Both breast cancer cell lines used in this study did not express HLA-E (Figure 6A). We compared the anti-tumor response of NKG2A^+^ vs. NKG2A^−^ NK cell subsets in the CD107a assays to test whether licensing by NKG2A can have a positive effect on NK cell degranulation against HLA-E negative breast cancer targets.

With MCF7 as target cells, NK cell degranulation was higher in the NKG2A^+^ subsets compared to the NKG2A^−^ subsets in all analyzed subsets without trastuzumab (matched and mismatched under normoxia and hypoxia), while degranulation in the presence of trastuzumab was comparable between NKG2A^+^ and NKG2A^−^ subsets (Figure 6B). In response to SKBR3, we also observed an increase in degranulation in the NKG2A^+^ NK cell population compared to the NKG2A^−^ population for all conditions without trastuzumab (Figure 6C). Since NK cell degranulation against SKBR3 was already so vigorously augmented by trastuzumab, a potential further increase due to NKG2A could not be detected (Figure 6C). Spontaneous NK cell degranulation without target cells was below 10% in all conditions (Figure S6). Overall, the NKG2A^+^ NK cell subsets responded slightly stronger than the NKG2A^−^ NK cells to the HLA-E negative cell lines MCF7 and SKBR3.

## 4. Discussion

With the aim to develop effective NK-cell-based therapies against breast cancer, we investigated the combination of the monoclonal antibody trastuzumab and KIR-ligand-mismatched donor NK cells to improve the responses against breast cancer in an immunosuppressive environment. We found that KIR-ligand-mismatched NK cell subsets degranulated stronger against breast cancer than their matched subsets and that trastuzumab activated all NK subsets when HER2 was overexpressed. Importantly, our observations were consistent in a hypoxic environment, emphasizing that the combination of reducing the activation threshold for NK activation by the selection of KIR-ligand-mismatched donors and maximizing NK cell activation with an ADCC-inducing antibody can potentiate the NK cell anti-breast cancer response.

In our experiments with cell lines, we used hypoxia to mimic one of the important factors in the TME. Severe hypoxia has namely been observed in the core of tumors from breast cancer patients and has been associated with metastasis formation and thereby with severity of disease [15]. Moreover, we and others showed that hypoxia can reduce effector functions of unactivated NK cells [20] and that NK cell activation with high-dose IL-2 could restore NK cell cytotoxicity against multiple myeloma [12]. Here, we report that the IL-2-activated NK cell also remained functional against breast cancer.

For SKBR3, we observed a small reduction in NK cell killing potential under hypoxia. This reduction could suggest an adaption to hypoxia in the SKBR3 target cells contributing to resistance to NK cells, e.g., by a reduction in activating ligands or enhanced expression of inhibitory ligands. Resistance could be acquired by the NK cells, e.g., via altered receptor expression, which would result in less efficient NK cell activation. In our previous study on hypoxia, we observed a minor decrease in expression of CD16 and NKG2D but not in any of the other common NK cell receptors [12]. Moreover, on multiple myeloma cell lines, we did not see a change in the expression of stress-induced activating ligands MICA/B and ULBP1/2 [12]. In the present study, we observed that expression of HLA class I was not altered by hypoxia and that IL-2-activated NK cells were potently degranulating under hypoxia. We therefore anticipate that NK cells were activated under hypoxia in this breast cancer setting and that resistance would mainly occur inside SKBR3 target cells. Baginska et al. also demonstrated that hypoxia-induced resistance was not caused by defective recognition of targets cells but by autophagy, leading to the breakdown of NK cells’ cytotoxic granules in hypoxic breast cancer cells [14]. To develop future strategies to improve the response in hypoxic tumors, it could be interesting to combine NK-cell-based strategies as proposed in this study with autophagy-reducing strategies such as chloroquine.

Although hypoxia is an important TME factor, our reductionist approach with cell lines did not take the full complexity of the TME into account, which may lead to an underestimation of the impact that hypoxia may have in combination with other TME factors. Our model with primary breast cancer samples showed considerable variability in sensitivity to NK cells between the different patients, illustrating the potential importance of other TME factors. The exposure of the primary breast cancer cells to the TME in patients is therefore an important advantage of our model, as it enabled us to assess the effects of TME-induced resistance mechanisms to NK cells more comprehensively. Examples of such cell TME resistance mechanisms are changes in the level of autophagy or expression levels of activating or inhibitory ligands on the tumor cells. However, a limitation of the model could be the relatively harsh digestion procedure, which could affect tumor cell viability as well as surface expression of activating ligands MICA and MICB [21], leading to underestimation of the contribution of these ligands. Moreover, it does not predict the direct effects that soluble TME factors and other tumor-associated cells can have on NK cells, which illustrates the necessity to develop more complex in vitro or in vivo models mimicking the multifactorial TME in patients to further evaluate the impact of the TME on NK cell efficiency.

In our study, trastuzumab enhanced NK cell degranulation much more vigorously against HER2-amplified targets compared to non-amplified targets in vitro, and it did not enhance NK cell functions against non-amplified primary breast cancer cells, suggesting that the HER2 expression level is important for the potential of the ADCC response. Our observations are in agreement with previous studies reporting that the effect of monoclonal antibodies such as daratumumab and trastuzumab is specific to target cells expressing high levels of antigen [17,22,23]. HER2 expression can be modulated through receptor internalization [24]. Recently, a study elegantly showed that HER2 internalization could be prevented by endocytosis inhibitors, resulting in an improved ADCC response [25]. In our study, HER2 could be detected after 4 h incubation with trastuzumab, and NK cells were potently degranulating in response to HER2 amplified cells, indicating that HER2 endocytosis cannot be the only factor limiting the ADCC response we observed.

We report a large discrepancy between a potent increase in NK cell degranulation against HER2-amplified target cells but a less pronounced effect on the actual killing of the target cells induced by trastuzumab. We have not previously noticed such a large discrepancy between degranulation and cytotoxicity. When using the same experimental setup with multiple myeloma cells as targets, we observed an ADCC effect with the anti-CD38 antibody daratumumab that was more similar to the degranulation effect [17]. Although further studies are required to unravel the mechanisms behind this observation, the strong degranulation with trastuzumab could indicate a very potent cytokine release by the NK cells. A profound release of cytokines, such as IFN-γ and TNF-α, by NK cells can boost the overall anti-tumor response by stimulating antigen presentation, Th1 polarization, and CD8 effector functions [26]. Studies evaluating the relation between cytokine production and degranulation (CD107a) on a single-cell level demonstrated that NK cells either produce cytokines, express CD107a, or do both upon activation with target cells [27]. Cytokine production has also been shown to occur in an HLA-dependent manner, as KIR-ligand-mismatched NK cells had higher intracellular IFN-γ levels than KIR-ligand-matched cells upon activation with L721.221 target cells [28]. The trastuzumab-induced degranulation of NK cells described in our study suggests that the combination of trastuzumab and IL-2-activated KIR-ligand-mismatched NK cells may also trigger a stronger production of cytokines, which could contribute to improved adaptive anti-tumor immunity.

In our study, we confirmed the functional relevance of HLA class I as an important inhibitory immune checkpoint for NK cell effector functions in breast cancer. As for many cancers, HLA class I expression can be partially or completely downregulated in breast cancer [29,30,31]. In our study, we mimicked the absence of HLA class I by using KIR-ligand-mismatched NK cells. In the presence of trastuzumab, KIR-ligand-mismatched NK cells remained the stronger degranulating subset against low HER2-expressing MCF7 cells, and when HER2 expression was high as in SKBR3, trastuzumab led to vigorous degranulation in all subsets, suggesting that the inhibitory ligand HLA class I matters less for the NK cell responses when trastuzumab is present. Muntasell et al. investigated predictive biomarkers for the response to trastuzumab treatment and found that patient stratification based on high HLA class I expression together with infiltrating NK cells improved prediction of better responses [32]. These data are in line with our results showing that NK cells can be effective against HLA class I^+^ tumors in combination with trastuzumab. Muntasell’s study also supports that both NK cells and T cells are major contributors to the anti-breast cancer response of HER2^+^ patients. 

Our results emphasize that selection of NK cell donors based on their KIR expression and HLA genotype can be an effective way to reduce inhibition in a setting with adoptive transfer of donor NK cells, which could be particularly useful for HER2-negative patients. However, since HER2 expression can be heterogeneously expressed within one tumor or be downregulated in response to trastuzumab treatment [33], KIR-ligand-mismatched NK cells may also be advantageous for HER2-positive patients treated with trastuzumab. Selection of KIR-ligand-mismatched donors is possible for patients that lack at least one of the three HLA epitope groups binding to inhibitory KIRs, which is the case for circa 70% of the population [34]. Although the here-evaluated KIR-ligand-mismatched subsets comprise a rather small percentage of total NK cells, KIR-ligand mismatching helps to reduce inhibition in these subsets, which can nonetheless be beneficial in a TME where many factors can limit NK cell anti-tumor responses. Our analysis indicated that the NKG2A^+^ KIR-ligand-mismatched NK cells performed as well as or slightly better than their NKG2A^−^ counterpart against HLA-E negative target cells. This observation implies that the NKG2A^+^ KIR-ligand-mismatched subsets can also be considered fully mismatched against HLA-E negative targets, which can significantly enlarge the mismatched NK cell population since NKG2A is expressed on 20–80% of NK cells. In breast cancer patients, HLA-E expression was detected in 20–50% of samples [30,35]. High HLA-E expression can, however, inhibit NK cell responses [36]. We did not have breast cancer cells available that expressed HLA-E. However, in a previous study with the same experimental setup, we showed that high HLA-E levels in multiple myeloma cells inhibited NK cells, while low HLA-E levels were not sufficient to do so [36]. The inhibitory potential of HLA-E has been established in multiple tumor models [37]. Therefore, it seems likely that high HLA-E expression will also limit NK cell anti-breast-cancer responses, and in those cases, blocking antibodies such as the anti-NKG2A antibody monalizumab might need to be considered. It would be relevant to further evaluate the additive effect of this approach on primary breast cancer with high levels of HLA-E.

Based on our results, we envision that alloreactive donors should be selected for NK cell-based therapies against HLA class I+ breast cancer. Multiple clinical trials showed that infusion of alloreactive NK cells is well tolerated when combined with lymphodepleting chemotherapy to suppress the host’s immune response. It needs to be assessed whether KIR-ligand mismatching can further enhance trastuzumab-induced NK cell degranulation in a setup that better represents the complex TME where many factors can limit NK cell anti-tumor responses. Genetic manipulation of NK cells could be a second strategy to limit inhibitory signaling via HLA, which could be done by CRISPR/CAS9-mediated knock-out of inhibitory receptors such as NKG2A and KIR. Another attractive strategy could be temporarily reducing expression levels of the inhibitory receptors via silencing RNAs. Given the critical role of NKG2A and KIR in NK cell licensing, transient reduction of receptor expression may be especially relevant when full receptor knock-outs negatively influence NK cell potency.

## 5. Conclusions

In this study, we showed that IL-2-activated NK cells can mediate anti-breast cancer responses under hypoxic conditions. Our data also illustrated the relevance of HLA class I as an inhibitory immune-checkpoint for NK cells in breast cancer, prompting follow-up studies to enhance NK cell responses by the combination of ADCC-triggering antibodies and strategies to interfere with the interaction between HLA class I and KIR/NKG2A. Here, we demonstrated two strategies, ADCC-triggering by the anti-HER2 antibody trastuzumab and selection of KIR-ligand-mismatched NK cell donors, to induce strong NK cell degranulation. Other strategies to interfere with HLA class I inhibition could include monoclonal antibodies that block inhibitory receptors on NK cells, such as monalizumab, or genetically modified NK cells to reduce expression of inhibitory NK cell receptors, as this would reduce the threshold for NK cell activation and may contribute to the development of curative immunotherapeutic strategies for breast cancer patients.

## Data Availability

The data presented in this study are available from the corresponding author upon reasonable request.

## Supplementary Materials

The following are available online at https://www.mdpi.com/article/10.3390/cancers13133232/s1, Figure S1: Anti-tumor efficacy of NK cell donors against the control target cell line K562, Figure S2: Titration of trastuzumab antibody with target cell line SKBR3, Figure S3: Gating strategy for the cytotoxicity assays with SKBR3 and K562 as target cells, Figure S4: NK cell degranulation depicted per KIR receptor in response to MCF7 and SKBR3, Figure S5: NK cell degranulation of NKG2A^−^ KIR-ligand-matched and mismatched NK cells depicted in response to K562 and depicted as fold-change compared to matched NK cells, Figure S6: NKG2A and KIR subset analysis of spontaneous degranulation in NK cells without target cells.
