# Supplementary material for: ADCC-Inducing Antibody Trastuzumab and Selection of KIR-HLA Ligand Mismatched Donors Enhance the NK Cell Anti-Breast Cancer Response
Source: Cancers (Basel). 2021 Jun 28;13(13):3232. doi: 10.3390/cancers13133232 (PMC8268223; doi:10.3390/cancers13133232)
Supplement: Supplementary file 1 [file cancers-13-03232-s001.zip › cancers-1244387-supplementary.pdf]

cancers

MDPI

Supplementary Materials: ADCC-Inducing Antibody Trastuzumab and Selection of KIR-HLA Ligand Mismatched Donors Enhance the NK Cell Anti-Breast Cancer Response

Femke A. I. Ehlers, Nicky A. Beelen, Michel van Gelder, Tom M. J. Evers, Marjolein L. Smidt, Loes F. S. Kooreman, Gerard M. J. Bos and Lotte Wieten

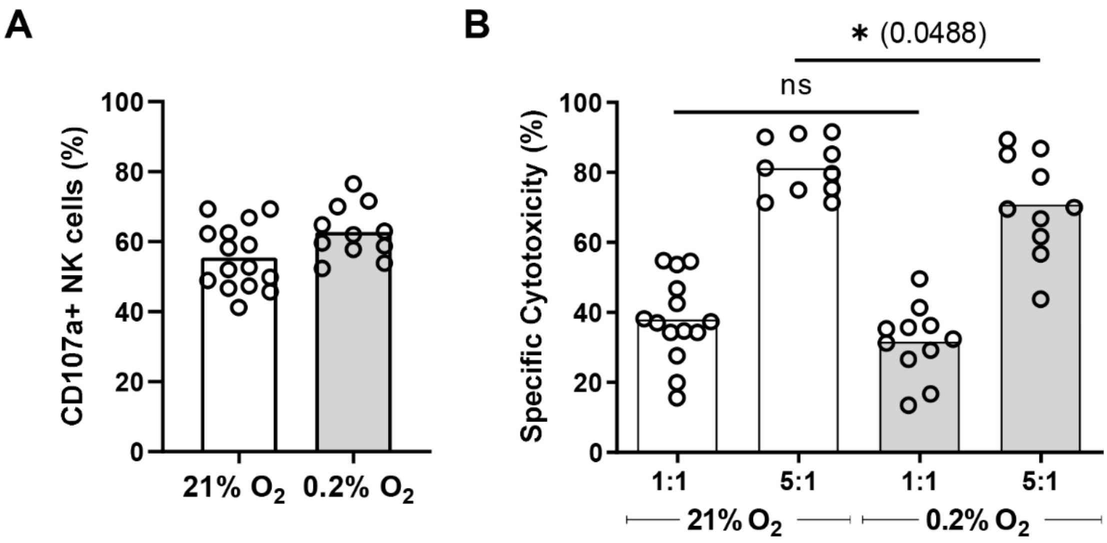

Figure S1. Anti-tumor efficacy of NK cell donors against the control target cell line K562. The HLA class I-negative cell line K562 was included as control target cells to test the NK cell killing potential of each donor. The degranulation (CD107a) and cytotoxicity assays were performed in the same way as described in Figure 1: IL-2 activated NK cells were co-cultured with the target cell line K562 for 4 h either with 21% O2 or 0.2% O2. Assays were measured by flow cytometry. (**A**) CD107a assays were performed in 1:1 E:T ratios and % of CD107a+ NK cells are shown per donor. (**B**) Cytotoxicity assays were done in 1:1 or 5:1 E:T ratios and dead target cells are shown as % of specific cytotoxicity. Each dot represents the average of duplicates from one NK cell donor and the bar height indicates the mean. * $p < 0.05$, ns = not significant.

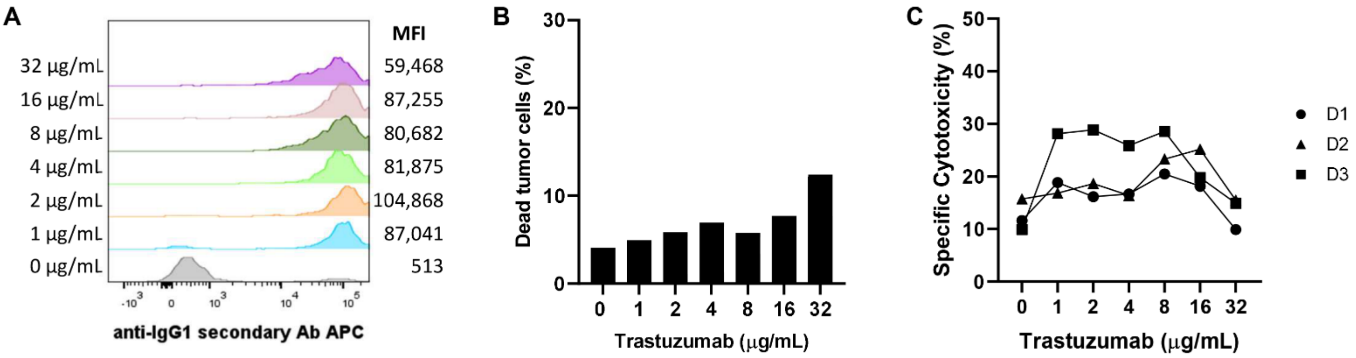

Figure S2. Titration of Trastuzumab antibody with target cell line SKBR3. SKBR3 cells were incubated with 0–32 µg/mL Trastuzumab for 30 min before either medium or IL-2 activated NK cells in a 1:1 E:T ratio were added for 4 h at 21% O_2. Analysis was performed by flow cytometry. (**A**) Binding of Trastuzumab to SKBR3 was detected by indirect staining with anti-IgG1 secondary antibody. (**B**) The spontaneous cell death of SKBR3 is reported as percentage of dead tumor cells. (**C**) Specific cytotoxicity of SKBR3 is shown. Each symbol represents one donor and the average of duplicates is depicted per donor.

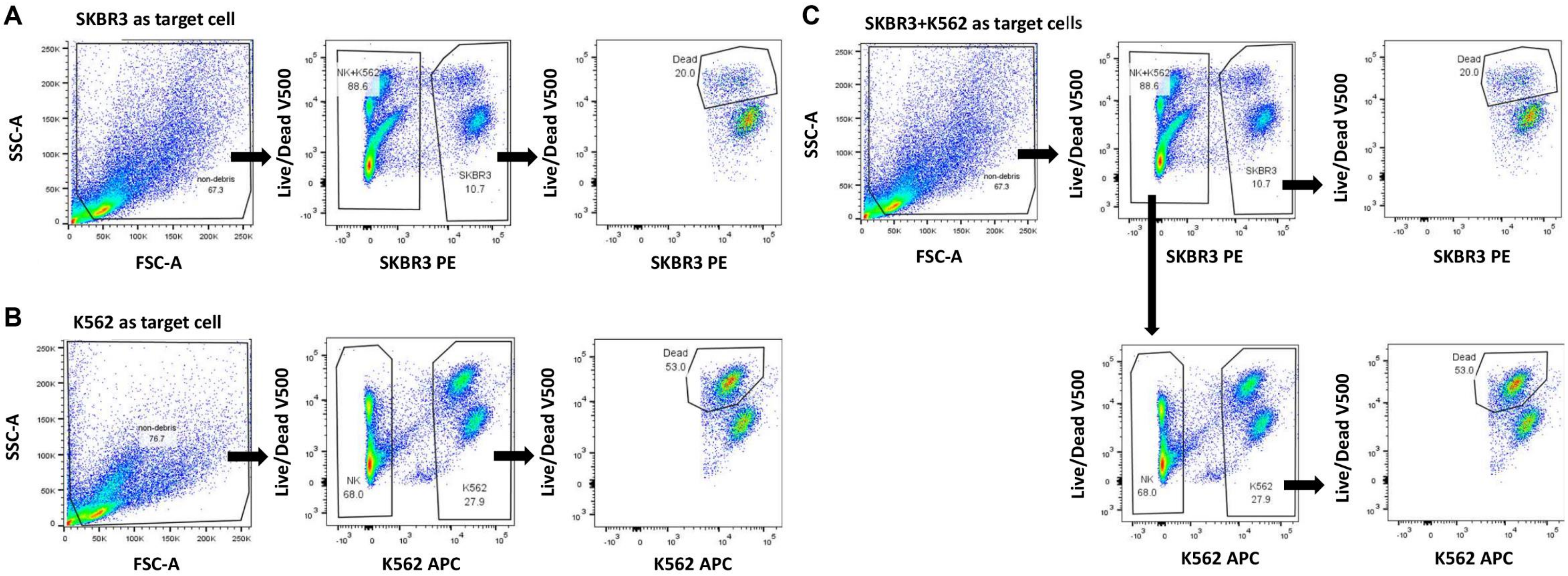

Figure S3. Gating strategy for the cytotoxicity assays with SKBR3 and K562 as target cells. First, debris was gated out, followed by gating on tumor cells that were labeled with a dye and subsequently dead tumor cells were gated. Representative example is shown for SKBR3 (**A**), K562 (**B**), SKBR3 and K562 combined (**C**).

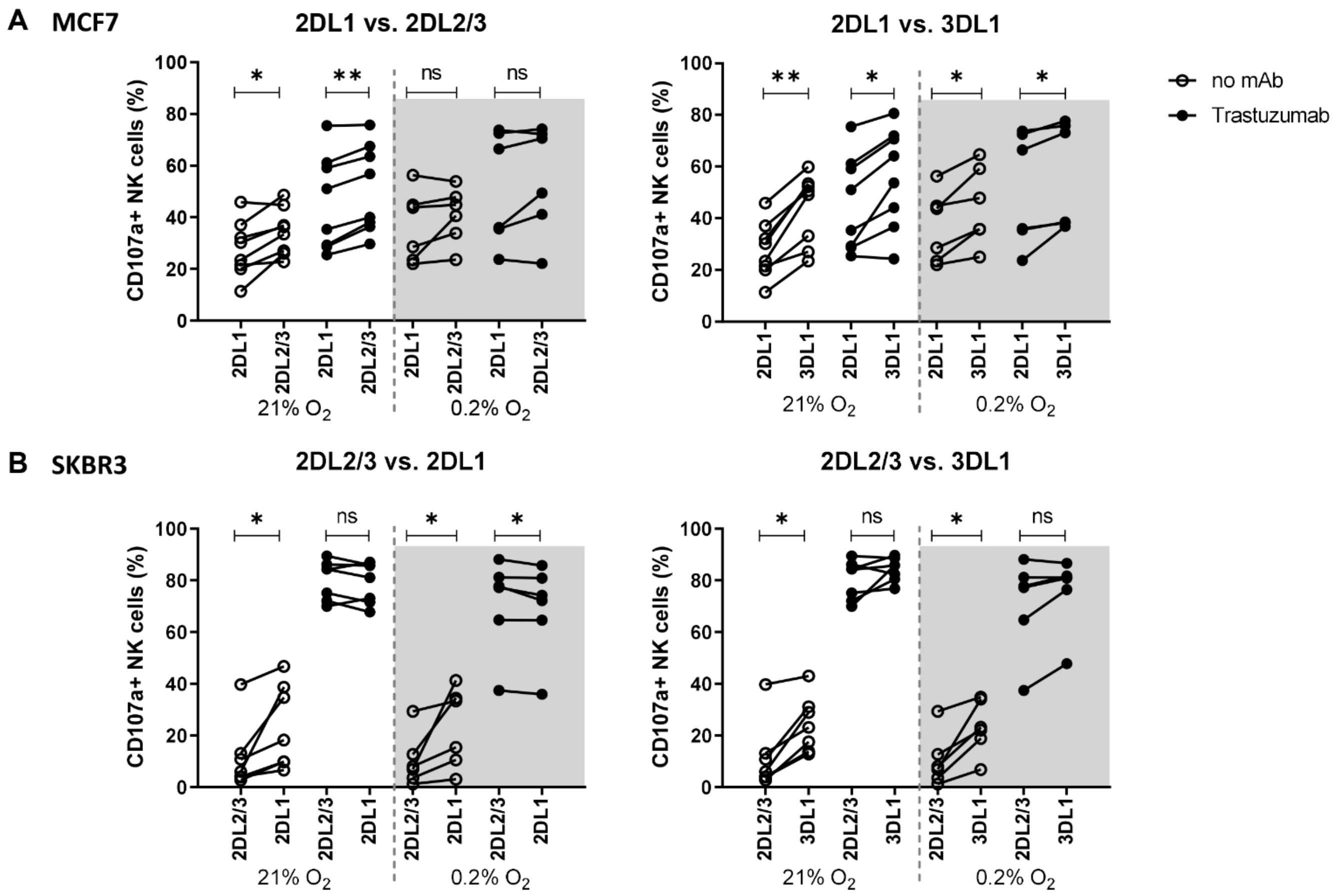

Figure S4. NK cell degranulation depicted per KIR receptor in response to MCF7 and SKBR3. Degranulation assays were performed by co-culturing NK wells either with MCF7 or SKBR3 target cells with or without Trastuzumab for 4 h at 21% O_2 or 0.2% O_2. NK cells that expressed one of the KIR receptors 2DL1, 2DL2/3, 3DL1 were selected by gating and the degranulation (% CD107a) is presented for each subset in response to MCF7 (**A**) or SKBR3 (**B**). Each dot represents the average of duplicates from one NK cell donor. * $p < 0.05$, ** $p < 0.01$, ns = not significant.

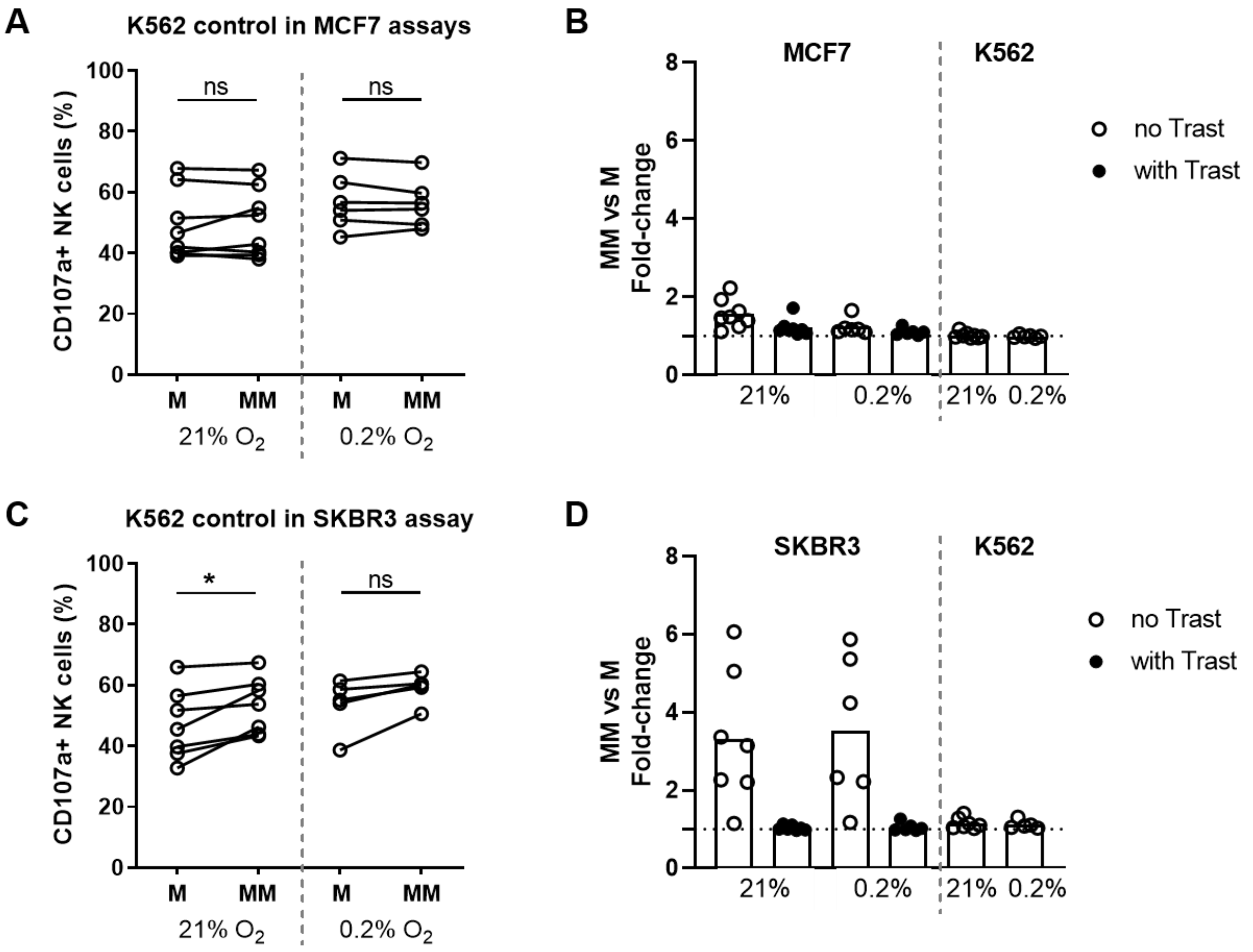

Figure S5. NK cell degranulation of NKG2A⁻ KIR-ligand matched and mismatched NK cells depicted in response to K562 and depicted as fold-change compared to matched NK cells. (**A**,**C**) The degranulation potential of the same KIR-ligand matched (M) and -mismatched (MM) NK cell subsets that were determined in response to MCF7 and SKBR3 in Figure 4 were analyzed for the HLA-negative control cell line K562. The fold-change in degranulation of the mismatched subset compared to the matched subset was calculated for MCF7 and K562 control (**B**) and SKBR3 and K562 control (**D**) where a value of 1 indicates no change. Each dot represents the average of duplicates from one NK cell donor. * $p < 0.05$, ns = not significant.

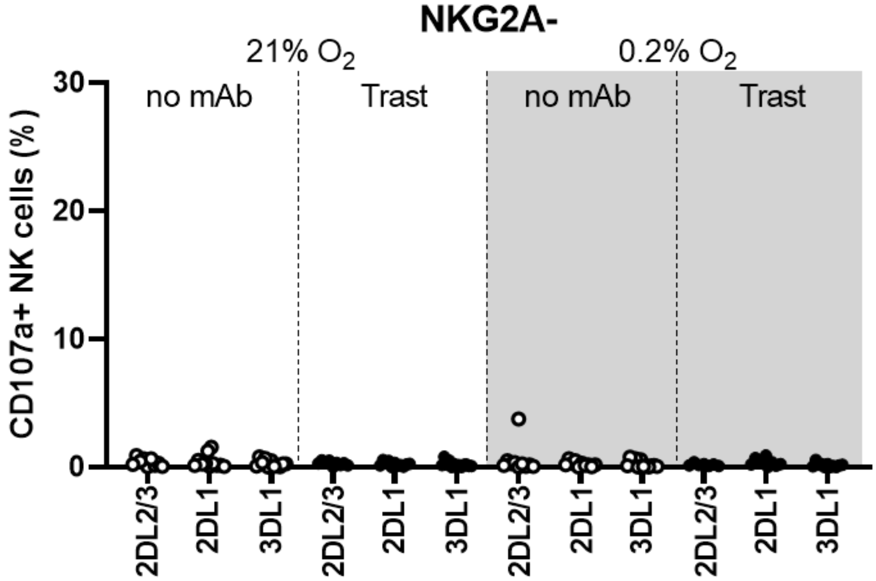

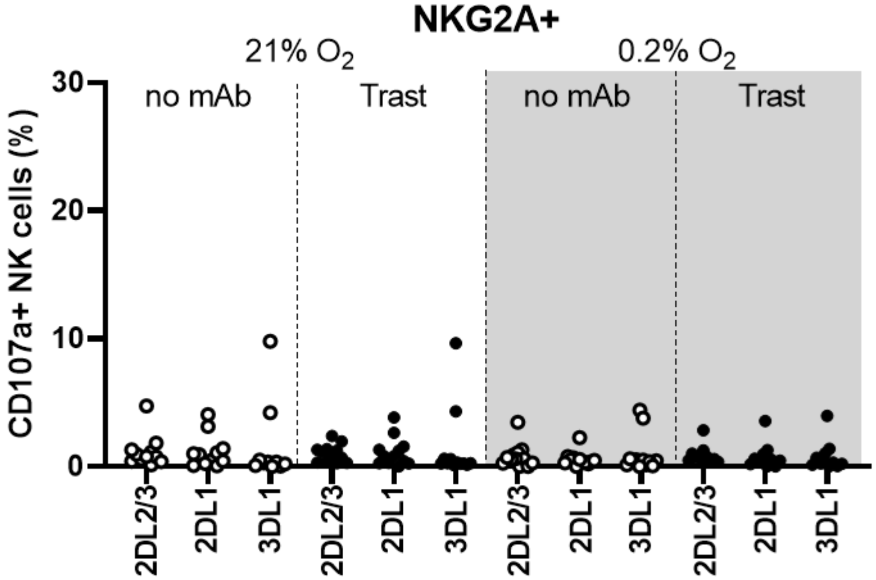

Figure S6. NKG2A and KIR subset analysis of spontaneous degranulation in NK cells without target cells. NK cells were cultured with or without Trastuzumab for 4 h at 21% O_2 or 0.2% O_2. By flow cytometry analysis, NK cells were grouped in NKG2A$^-$ subsets (above) and NKG2A$^+$ subsets (below) and the spontaneous degranulation of NK cells is presented as % CD107a+ cells for the three inhibitory KIRs 2DL2/3, 2DL1 and 3DL1. Each dot represents one NK cell donor.
